# Acute kidney injury in cardiac surgery patients receiving hydroxyethyl starch solutions

**DOI:** 10.1186/s13054-015-0859-z

**Published:** 2015-05-05

**Authors:** Ole Bayer, Konrad Reinhart

**Affiliations:** Department of Anaesthesiology and Intensive Care, Jena University Hospital, Erlanger Allee 101, 07747 Jena, Germany

In a retrospective study by a Canadian team [1], pentastarch infusion was a dose-related independent risk factor for acute kidney injury (AKI) after cardiac surgery. In a new retrospective cardiac surgery study by that team [2], 83% of patients received hydroxyethyl starch (HES) 130/0.4. For unexplained reasons, 25 to 43% of patients received both HES 130/0.4 and pentastarch.

The team 'hypothesized that both synthetic starches and albumin-containing solutions are independently associated with AKI following cardiac surgery in a dose-dependent fashion'. However, they focused on albumin and never thoroughly evaluated HES-related AKI. Although univariate analyses were reported, propensity matching according to either HES 130/0.4 or pentastarch administration was omitted. Systematic allocation of low-risk patients to HES could have masked an association with AKI in the univariate analyses. Consequently, the study is misleading, since it suggests that albumin is associated with AKI while HES is not.

We described a prospective study in 6,478 consecutive cardiac surgery patients [3]. With propensity matching, predominant use of HES 130/0.4 was associated with increased utilization of renal replacement therapy: odds ratio 1.46 and 95% confidence interval (CI) 1.08 to 1.97. Furthermore, in a meta-analysis of 15 randomized trials evaluating perioperative HES administration, including five in cardiac surgery, renal replacement therapy was increased by HES solutions as a class with relative risk 1.44 and CI 1.04 to 2.01 and by HES 130/0.4 in particular (relative risk 1.47, CI 1.02 to 2.12) [4]. Based on these results and other currently available data, complete avoidance of HES solutions such as HES 130/0.4 has been recommended [5].

## Abbreviations

AKI: Acute kidney injury
CI: Confidence interval
HES: Hydroxyethyl starch
