# Supplementary material for: Acute kidney injury in cardiac surgery patients receiving hydroxyethyl starch solutions
Source: Crit Care. 2015 May 5;19(1):209. doi: 10.1186/s13054-015-0859-z (PMC4419457; doi:10.1186/s13054-015-0859-z)
Supplement: Related texts

# Reply: Authors’ response

**Authors:** Josée Bouchard, Anne Julie Frenette, Stéphan Troyanov, David R Williamson

We agree with Bayer and Reinhart that the administration of HES solutions should be avoided, based on results from randomized trials [6,7]. Indeed, we had acknowledged in the article that HES solutions are an independent risk factor for AKI [2]. In contrast to what the authors mentioned, our propensity score included the percentage and dose of HES administered [2]. In our study, the risk of AKI appeared higher with albumin than with HES (Figure three in [2]). Given a recent increase in albumin use in our institution in light of recent HES publications, we felt prudent to test whether this finding was artifactual.

Over the past decade, several studies have been published on the timing [8], duration, type [6], and amount of fluid [9] to be given in critically ill patients. However, the best approach regarding fluid resuscitation is still uncertain and many other questions remain unanswered. When, how much, how fast, and how long should we administer which type of fluid to optimize cardiac output, while minimizing potential resultant fluid accumulation, tissue edema and consequent organ dysfunction? As critically ill patients are a heterogeneous population, a treatment may be beneficial to one subgroup of patients but harmful to another. Our study results do not suggest that albumin should never be administered in cardiac surgery patients. Further studies are needed to define the best type of fluid (balanced crystalloids, isotonic saline and albumin), optimal amount, timing and duration in *a priori* defined critically and non-critically ill populations.
